# Impact of Four Rounds of Mass Drug Administration with Dihydroartemisinin–Piperaquine Implemented in Southern Province, Zambia

**DOI:** 10.4269/ajtmh.19-0659

**Published:** 2020-07-02

**Authors:** Thomas P. Eisele, Adam Bennett, Kafula Silumbe, Timothy P. Finn, Travis R. Porter, Victor Chalwe, Busiku Hamainza, Hawela Moonga, Emmanuel Kooma, Elizabeth Chizema Kawesha, Mulakwa Kamuliwo, Joshua O. Yukich, Joseph Keating, Kammerle Schneider, Ruben O. Conner, Duncan Earle, Laurence Slutsker, Richard W. Steketee, John M. Miller

**Affiliations:** 1 Department of Tropical Medicine, Center for Applied Malaria Research and Evaluation, Tulane University School of Public Health and Tropical Medicine, New Orleans, Louisiana;; 2 Malaria Elimination Initiative, Global Health Group, University of California San Francisco, San Francisco, California;; 3 PATH Malaria Control and Elimination Partnership in Africa (MACEPA), Lusaka, Zambia;; 4 Institute for Medical Research and Training, University Teaching Hospital, Lusaka, Zambia;; 5 National Malaria Elimination Centre, Zambia Ministry of Health, Lusaka, Zambia;; 6 Zambia Ministry of Health, Southern Provincial Health Office, Choma, Zambia;; 7 PATH MACEPA, Seattle, Washington

## Abstract

Over the past decade, Zambia has made substantial progress against malaria and has recently set the ambitious goal of eliminating by 2021. In the context of very high vector control and improved access to malaria diagnosis and treatment in Southern Province, we implemented a community-randomized controlled trial to assess the impact of four rounds of community-wide mass drug administration (MDA) and household-level MDA (focal MDA) with dihydroartemisinin–piperaquine (DHAP) implemented between December 2014 and February 2016. The mass treatment campaigns achieved relatively good household coverage (63–79%), were widely accepted by the community (ranging from 87% to 94%), and achieved very high adherence to the DHAP regimen (81–96%). Significant declines in all malaria study end points were observed, irrespective of the exposure group, with the overall parasite prevalence during the peak transmission season declining by 87.2% from 31.3% at baseline to 4.0% in 2016 at the end of the trial. Children in areas of lower transmission (< 10% prevalence at baseline) that received four MDA rounds had a 72% (95% CI = 12–91%) reduction in malaria parasite prevalence as compared with those with the standard of care without any mass treatment. Mass drug administration consistently had the largest short-term effect size across study end points in areas of lower transmission following the first two MDA rounds. In the context of achieving very high vector control coverage and improved access to diagnosis and treatment for malaria, our results suggest that MDA should be considered for implementation in African settings for rapidly reducing malaria outcomes in lower transmission settings.

## INTRODUCTION

Over the past decade, Zambia has made substantial progress against malaria by scaling up national coverage of long-lasting insecticide-treated nets (LLINs), targeted indoor residual spraying (IRS), and improved access to diagnosis and treatment.^1^ The Zambia Ministry of Health recently set the ambitious goal of eliminating malaria by 2021.^2^ Across Southern Province, in combination with improved surveillance at health centers with the District Health Information System 2 (DHIS2) software platform, the Zambia National Malaria Control Centre scaled up community-based diagnosis and treatment through trained community health workers (CHWs) with surveillance from 2011 through the latter half of 2014. Community health workers were provided malaria rapid diagnostic tests (RDTs) for use in suspected malaria patients, as well as artemether–lumefantrine (AL) for treatment of those with confirmed infection, with the goal of subnational elimination in this area.

Whereas LLINs, IRS with pyrethroids, and improved access to case management had been used across Zambia, starting in 2011–2014, a scaled intervention package was introduced in the higher transmission districts in Southern Province along Lake Kariba. This consisted of universal access to LLINs, targeted IRS coverage with highly effective Actellic^®^ 300CS (Syngenta Global, Basel, Switzerland), and improved access to diagnosis and treatment in the community with the addition of CHWs as part of a national CHW expansion effort. In this context, 60 health facility catchment areas (HFCAs), stratified by lower and higher transmission areas, took part in a community-randomized controlled trial to assess the impact of community-wide mass drug administration (MDA) with the long-acting antimalarial dihydroartemisinin–piperaquine (DHAP) and focal MDA (fMDA) at the household level with DHAP, compared with a control of no mass treatment.^3^ Following two rounds of MDA and fMDA, parasite prevalence during peak transmission (April and May) declined markedly across the entire study area from 31.3% at baseline in 2014 8.4% in 2015, irrespective of mass treatment exposure or baseline prevalence. In the higher transmission strata, the reductions were seen in all intervention and control groups, with limited evidence of MDA having a substantial impact on infection prevalence. In the lower transmission strata, malaria parasite prevalence declined from 7.7% at baseline to 0.5% after the first two MDA rounds, an 87% larger decline than seen in the control group.

Here, we present the trial results of all four rounds of MDA and fMDA with DHAP implemented between December 2014 and February 2016, as compared with a control of no mass treatment (standard of care) within the context of the scaled intervention package implemented throughout the trial area. In doing so, overall trends in the population coverage of the mass treatment rounds, as well as malaria outcomes, over the course of the 2-year trial are presented. Trends in the primary components of the scaled intervention package (LLINs, IRS, and access to diagnosis and treatment) before and during the trial are also presented.

## METHODS

The full protocol for this trial, as well as the trial methods and results after the first two MDA rounds, has been published elsewhere.^3,4^ Ethical approval was obtained from the institutional review boards at Tulane University, Western Institutional Review Board (for PATH), and the University of Zambia. The trial was also approved by the Zambia Medicines Regulatory Authority and the National Health Research Authority for conduct in Zambia. Written informed consent was obtained from each participant before enrollment in the trial. The trial is registered with ClinicalTrials.gov (NCT02329301).

### Study design, study site, and participants.

A community-randomized controlled trial was carried out in 60 HFCAs from May 2014 to May 2016 to evaluate the impact of four rounds (two sequential rounds in each, the first year and the second year) of the mass treatment interventions with DHAP at reducing the study end points. The trial area was selected because it contains areas of historically higher and lower transmission, allowing us to test the mass treatment strategies across a range of transmission. As such, the trial area was equally stratified a priori into higher (≥ 10% parasite prevalence in children younger than 5 years; *n* = 30 HFCAs) and lower (< 10% parasite prevalence in children younger than 5 years; *n* = 30 HFCAs) transmission strata at randomization.

**Figure 1.**
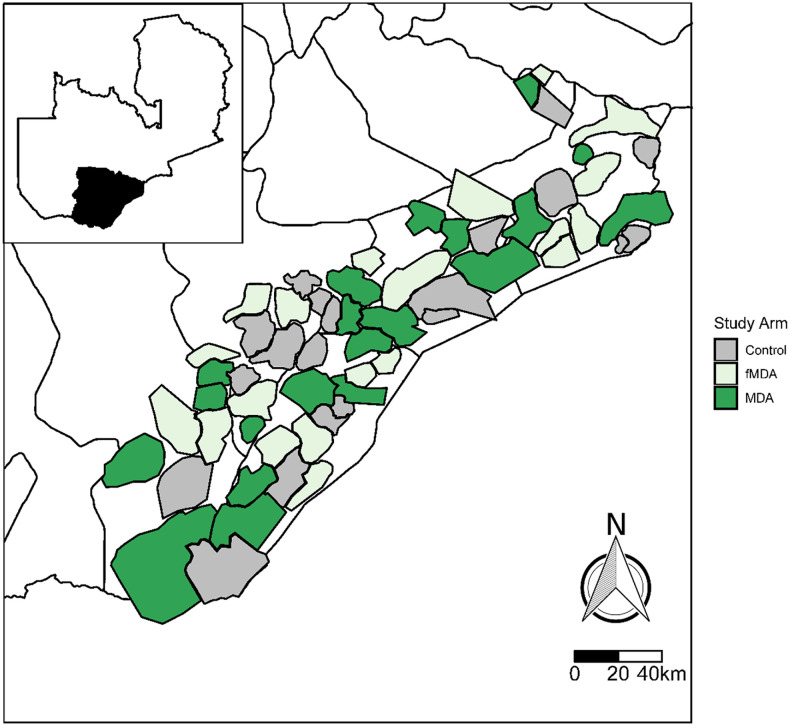
Map of 60 health facility catchment areas included in the mass treatment trial.

The trial was conducted in an area within 10 districts along Lake Kariba in Southern Province, Zambia (Figure 1). All households in the study site (approximately 330,000 people in 56,000 households, a third of which reside in each of the three exposure arms) were geographic information system (GIS)-enumerated in 2013 and 2014 before the trial. Malaria transmission in the study site is seasonal, with the peak occurring in April–May of each year at the end of the annual rainy season (December to April), ranging from greater than 50% parasite prevalence along the lake to less than 10% among inland populations at higher elevation.^5^

### Randomization.

Health facility catchment areas served as the unit of randomization. The 60 HFCAs were randomly assigned via a computer algorithm (unblinded) to the MDA, fMDA, or a control of no mass treatment using the random allocation rule to ensure each exposure group was assigned 20 HFCAs, within lower and higher transmission strata.^6^

### Standard of care, interventions, and study time line.

The standard of care for malaria across Zambia consists of vector control with either LLINs or IRS, as well as diagnoses (with either an RDT or microscopy) of all suspected cases presenting to the health system, with all positive individuals treated with the first-line treatment AL.^7^ However, under the status quo standard of care across most of Zambia, vector control coverage occasionally lapses because of the timing of intervention campaigns, whereas access to diagnosis and treatment can be challenging because of difficulty in accessing services at distant health facilities. Throughout the trial area, a scaled intervention package, with no mass treatment, served as the standard of care. This intervention package focused on ensuring there was universal coverage of LLINs combined with targeted use of Actellic 300CS for IRS, high-quality surveillance and reporting, and improved access to diagnosis and treatment through the expansion of CHW-based community case management (CCM). To achieve this, a comprehensive net campaign was carried out in the trial area in August 2014, just before the first mass treatment round. Two rounds of IRS Actellic were carried out in the trial area just before the rainy season in November of 2014 and 2015, and were targeted to sprayable houses in the highest risk areas with the goal of reaching 50% household coverage across the trial area. Starting in the latter half of 2014 before the start of MDA campaigns, CCM was scaled up throughout the trial area by placing CHWs who were specifically trained and equipped with RDTs and AL to diagnose individuals with suspected malaria and treat those found to be positive. Where logistically feasible, reactive case detection was also carried out by CHWs, consisting of RDT-testing all community members within a 140-m radius of a confirmed index case, with all positives treated according to the national guidelines.

**Figure 2.**
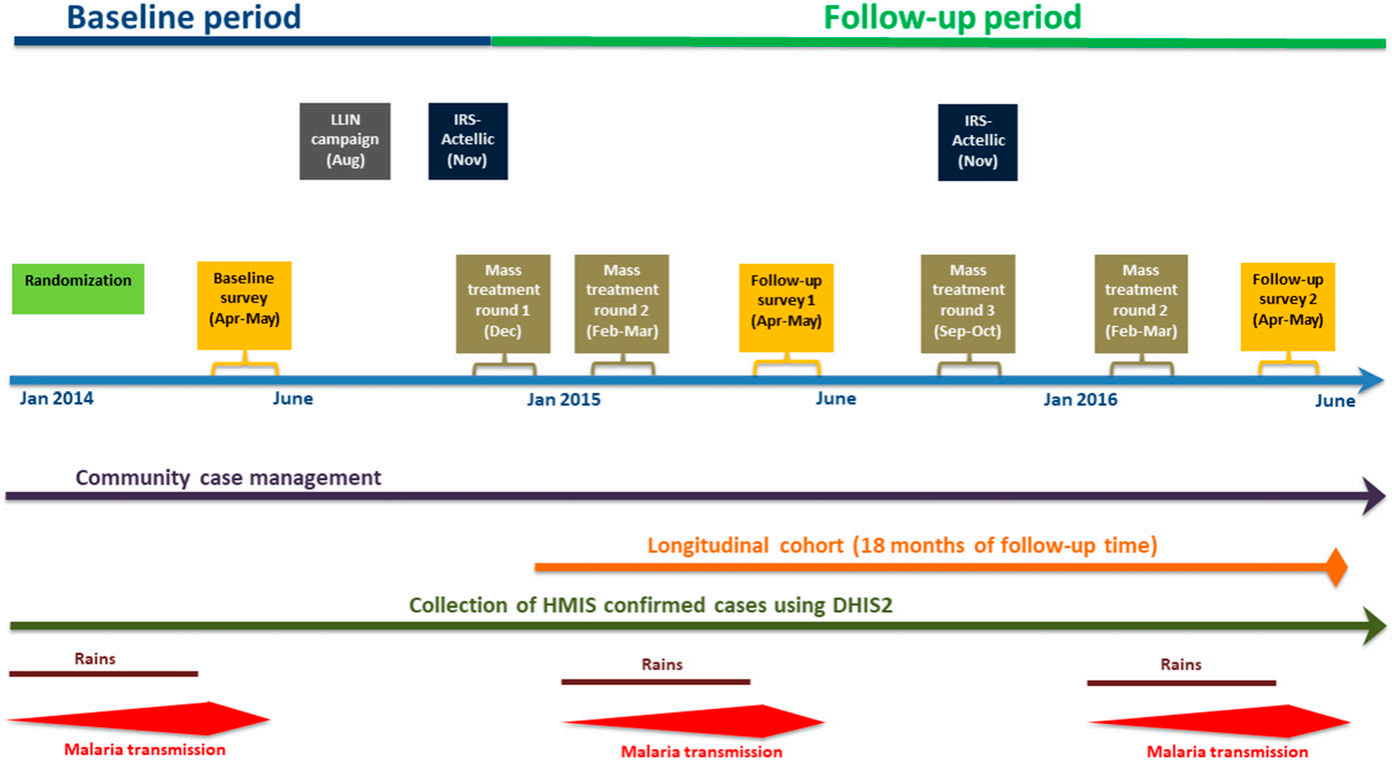
Trial time line of major activities. This figure appears in color at www.ajtmh.org.

All individuals in MDA- or fMDA-designated intervention clusters were tested for a *Plasmodium falciparum* parasite infection during each mass treatment campaign using an RDT (SD Bioline^®^ Malaria Antigen *P. falciparum* for detecting the histidine-rich protein two antigen, Standard Diagnostics Inc., Gyeonggi-do, Republic of Korea). Mass drug administration consisted of offering all eligible individuals DHAP, irrespective of an RDT result. Focal MDA consisted of offering DHAP to all eligible individuals who resided in a household where anyone tested positive by RDT. An age-appropriate 3-day course of DHAP (Sigma Tau, brand name Eurartesim^®^, Rome, Italy) was used among all eligible and consenting individuals during each mass treatment round, according to the manufacturer’s and national treatment guidelines for treating malaria (see Supplemental Information Table 1 for age-based DHAP dosing guidelines).^7^ The first and last doses of each 3-day course were given under directly observed therapy to the extent possible. The timing of the four rounds went as follows: round 1 took place just before the rainy season in December 2014, round 2 occurred during the rainy season in February–March 2015, round 3 occurred during the dry season in October 2015, and round 4 occurred during the rainy season in February 2016 (Figure 2). The rounds were intended to be implemented at the end of each dry season or just at the beginning of the rainy season. Timing of the interventions varied slightly between the first and second years because of logistical issues. In both the treatment arms, children aged < 3 months and pregnant women in their first trimester were excluded from receiving DHAP according to the manufacturer recommendations; they were instead offered the appropriate dose of antimalarial treatment according to the Zambian national policy if RDT positive.

Extensive community engagement activities were undertaken before and during the first and second sets of mass treatment campaigns. As described elsewhere,^8^ the community engagement strategy included district consultative meetings, local chiefs’ orientation, village meetings, drama performances, community radio messages, visual-based print materials for household interactions, public address announcements, and the use of CHWs, religious leaders, teachers, and neighborhood health committees as avenues of dissemination.

The control group received the standard of care package that was implemented across the entire study area, as described earlier, but did not receive any mass treatment. All individuals with severe illness of any kind were referred to the nearest health facility and were not included in the study.

### Study end points.

The primary study end point was malaria parasite infection prevalence among children aged < 6 years by RDT, with secondary end points consisting of cumulative infection incidence among all ages by PCR and RDT, and confirmed malaria case incidence among all ages. Cumulative infection incidence was defined as the number of parasite infections by PCR (all ages ≥ 3 months old) in a longitudinal cohort divided by their total time of exposure. Confirmed malaria case incidence rate was defined as the number of outpatient and community-based laboratory-confirmed malaria cases per 1,000 population per year.

### Measurement of malaria program coverage and potential confounding factors.

Long-lasting insecticide-treated net coverage was defined as the proportion of households with ≥ 1 LLIN. Indoor residual spraying coverage was defined as the proportion of households reporting their domicile was sprayed by the national program within the last 12 months. Both were measured using a standardized questionnaire asked to the head of the household during three household surveys conducted during the high-transmission season (April and May) at baseline in 2014, after mass treatment rounds 1 and 2 in 2015, and after mass treatment rounds 3 and 4 in 2016 (Figure 2). As described elsewhere,^4^ each survey consisted of a simple random sample of households within each of the 60 HFCAs from a complete enumeration (with geo-coordinates) of households in early 2014 that served as the sampling frame. Age, gender, recent history of fever, treatment seeking, and malaria treatment for all individuals in the household, as well as household socioeconomic status, were ascertained from a standardized questionnaire during each of the three household surveys. In addition to treatment seeking ascertained from respondent recall from the survey questionnaire, an indicator of access to malaria diagnosis and treatment at each survey round was estimated by the Euclidean distance of each surveyed household and either the closest health facility or CHW; geo-coordinates were available for all surveyed households and health facilities. The geo-coordinates of CHW residence were updated each year as their involvement in CCM for malaria was scaled-up across Southern Province. Monthly total rainfall was obtained from the Climate Hazards Group InfraRed Precipitation data. The enhanced vegetation index (which represents a proxy measure of an area’s propensity to harbor mosquitoes) was ascertained from publicly available satellite data. The methods used to assess population coverage of the mass treatment rounds, adherence to DHAP, and acceptability of MDA are presented elsewhere.^8–10^

### Measurement of malaria parasite infection prevalence.

*Plasmodium falciparum* parasite infection prevalence in children was measured using RDTs during the peak transmission season by the household surveys conducted at baseline (2014), follow-up 1 (2015), and follow-up 2 (2016), as described earlier and in detail elsewhere.^4^ A sample size of 2,820 children in each of the three survey rounds was sought to detect a 50% reduction in infection prevalence from the baseline to the first follow-up survey, with 80% power taking into account the cluster randomization with coefficients of variation of 0.41 and 0.31 in lower and higher transmission strata, respectively.^11^

### Measurement of cumulative infection incidence.

Details of the methods for the longitudinal cohort for ascertaining cumulative infection incidence are described elsewhere.^3,4,12^ In summary, cumulative infection incidence was measured among a target sample size of 2,250 individuals aged ≥ 3 months enrolled in a cohort at the time of the first mass treatment intervention (December 2014). At enrollment, all participants in the MDA and fMDA arms were presumptively cleared of any infection with DHAP that coincided with the first mass treatment round. Participants in the control group who were RDT positive at enrollment were cleared with AL at this same time. Participants were then visited each month for 17 follow-up months (January 2015 through May 2016). At each visit, an RDT and dried blood spot were collected. All participants at monthly follow-up visits with a positive RDT received AL. Participants were asked during each monthly visit about any fevers and/or treatment for malaria that occurred since the last cohort follow-up visit.

### Measurement of confirmed malaria case incidence.

Routine DHIS2 data on monthly confirmed outpatient malaria cases were ascertained from all 60 health facilities, and their CHWs, in the study area from January 2012 through May 2016. Confirmed case counts were standardized by the estimated midyear populations of each HFCA to obtain the confirmed malaria case incidence rate per 1,000 population.

### Statistical analysis.

All analyses were based on intention-to-treat where all individuals were assumed to receive the exposure assigned to their HFCA during randomization. All analyses were stratified a priori by the lower and higher transmission strata. The community-randomized controlled trial study design was accounted for by including the cluster (HFCA) as a random effect in all analyses.

The effect of MDA/fMDA exposure compared with control for malaria infection prevalence in children was estimated posttest for the 2015 and 2016 follow-up surveys separately, as well as pooled across the 2015 and 2016 surveys, using a crude odds ratio in a bivariate logistic regression model. A secondary model, defined a priori, adjusted for child age (in years), gender, household wealth from an asset index, rainfall, enhanced vegetation index, household elevation, and household protection by LLINs and IRS.

Incidence rate ratios (IRRs) from a random-effects negative binomial regression model were used to estimate the exposure effect of four rounds of MDA/fMDA, compared with the control group, on the PCR-determined cumulative infection incidence among cohort participants. Individuals who were present and tested at enrollment (baseline) and completed ≥ 3 months of the first 6 months of follow-up were included in the cohort analysis. Because of the timing of the mass treatment rounds and because their impact was expected to differ significantly between the rainy (high transmission) and dry (low transmission) seasons, the cohort analyses were stratified into the following three time segments: period 1 during the first rainy season post-enrollment from January through May 2015, period 2 during the dry season from April through November 2015, and period 3 during the second rainy season from December 2015 through May 2016. A second random-effects negative binomial model included adjustment for first month’s infection status, child age, gender, household wealth, rainfall, enhanced vegetation index, household elevation, and household protection by IRS.

We estimated the combined exposure effect of all four rounds of MDA/fMDA, compared with the control group, on monthly confirmed malaria case incidence using a negative binomial model, standardized by midyear catchment population estimates. The model controlled for the previous month’s cases, calendar month, rainfall, and enhanced vegetation index monthly anomalies. The analysis compared changes in confirmed malaria case incidence over a pooled baseline period from January through December 2013 and 2014 (pre-mass treatment) with a pooled post-mass treatment period from January through December 2015 and 2016. Because there were substantial differences between the three exposure groups (MDA, fMDA, and control group) in monthly confirmed malaria case incidence during the baseline period, a difference-in-differences (DiD) model was used to account for baseline case incidence, whereby the exposure effect represents the difference in the change in confirmed case incidence within the MDA and fMDA versus the control group, from the baseline to follow-up periods.

## RESULTS

Across all four mass treatment rounds, 65,040 households were visited and invited to partake in the MDA campaigns and 66,370 households were offered participation in the fMDA rounds (Table 1). A total of 268,502 courses of DHAP were administered to eligible and consenting individuals during the four MDA rounds, irrespective of the RDT result, and 65,319 courses of DHAP were administered to individuals in households with an RDT-positive resident during the four fMDA rounds. Data from passive surveillance of adverse events showed DHAP to have been well tolerated in this study.^13^ Data from the household survey and accompanying qualitative research indicated MDA was a widely acceptable intervention in the study communities.^8^ Overall, adherence to the 3-day DHAP treatment course was estimated to be 82.4% and 92.8% across all four MDA and fMDA rounds, respectively; adherence rates in the fMDA arm were higher than those in the MDA arm in all rounds.^10^ There was no evidence of treatment failure from DHAP.^10^ Household coverage of MDA across all four rounds was estimated to be 70.6%, ranging from 63.4% to 79.3%; coverage across all four fMDA rounds was similar at 71.4%, ranging from 62.2% to 76.6%. Coverage was lower for mass treatment rounds during the rainy season when access to some areas was difficult.^9^

**Table 1:** Program data and coverage from MDA and fMDA rounds, Southern Province, Zambia, 2014–2016

	Round 1 (December 2014)		Round 2 (February 2015)		Round 3 (October 2015)		Round 4 (February 2016)		Total	
	MDA	fMDA	MDA	fMDA	MDA	fMDA	MDA	fMDA	MDA	fMDA
Houses visited*	18,237	17,704	14,584	14,610	17,528	18,004	15,257	16,050	65,040	66,370
DHAp courses administered*	78,591	25,372	56,620	17,092	72,006	14,599	64,285	8,256	271,502	65,319
Adherence to all three DHAp courses (%)†	80.6 (71.7–87.2)	91.4 (86.7–94.7)	84.6 (76.0–90.5)	91.7 (87.4–94.7)	80.6 (71.1–87.6)	95.6 (92.4–97.5)	84.9 (75.4–91.2)	93.3 (88.1–96.4)	82.4 (74.5–88.2)	92.8 (89.9–95.0)
Household coverage (%)‡	79.3 (74.2–8.5)	75.3 (70.3–80.3)	63.4 (59.3–67.6)	62.2 (58.0–66.3)	76.3 (71.3–81.2)	76.6 (71.5–81.2)	66.4 (62.0–70.7)	68.3 (63.8–72.8)	70.6 (65.9–75.3)	71.4 (66.7–76.0)

DHAp = dihydroartemisinin–piperaquine; fMDA = focal MDA; MDA = mass drug administration.

* Ascertained from program records.

† Ascertained from household surveys.

‡ Estimated using capture–recapture of household lists from surveys and mass treatment rounds.

As previously reported, there were no differences among the MDA, fMDA, and control groups at baseline in child and household demographics, treatment seeking for fevers, intervention coverage, and climate (Table 2 and Supplemental Table 2).^3^ Household LLIN possession and use by children younger than 6 years did not differ between intervention and control groups post-baseline during the 2 years of the trial (Table 2). However, the use of LLINs by children increased significantly from 46.8% at baseline to 64.9% during the first year of the trial (Rao–Scott χ^2^ = 22.58, df = 1; *P* < 0.0001); there was no difference in rates of LLIN use between 2015 and 2016. As previously reported, IRS coverage at baseline was significantly higher in the fMDA (19.7%) and control groups (17.0%) than the MDA group (6.9%, Rao–Scott χ^2^ = 6.00, df = 2; *P* = 0.0497),^3^ although no differences between exposure groups were observed after baseline. Household IRS coverage significantly increased across all exposure groups from baseline (14.5%) to the first year of the mass treatment rounds 1 and 2 (36.7%, Rao–Scott χ^2^ = 24.05, df = 1; *P* < 0.0001); there were no significant differences between exposure groups in IRS coverage between 2015 and 2016.

**Table 2:** Intervention coverage changes across trial area, April–May 2014–2016, by exposure group

Intervention	Survey 2014 (baseline)			Survey 2015 (rounds 1–2)			Survey 2016 (rounds 3–4)		
	MDA	fMDA	Control	MDA	fMDA	Control	MDA	fMDA	Control
Sample sizes (*n*) households	857	850	866	769	749	806	940	911	913
Children	1,047	985	976	745	652	708	839	834	812
Vector control									
% Household ≥ 1 LLIN (95% CI)	70.25 (62.44–78.05)	73.18 (65.78–80.57)	75.29 (68.71–81.87)	81.01 (72.89–89.14)	79.44 (72.71–86.17)	85.98 (79.13–92.83)	77.23 (69.91–84.56)	76.40 (70.92–81.88)	78.75 (72.65–84.86)
% Children who slept under LLINs on the previous night (95% CI)	46.61 (37.05–56.17)	47.71 (40.54–54.89)	46.21 (39.49–52.92)	61.75 (52.64–70.85)	65.49 (55.97–75.01)	67.80 (58.33–77.26)	62.45 (55.12–69.79)	59.71 (51.59–67.83)	59.98 (54.44–65.51)
% Households with IRS in the past 12 months (95% CI)	6.88* (2.34–11.43)	19.65* (9.46–29.84)	16.86* (6.13–27.59)	33.29 (18.99–47.59)	36.85 (20.65–53.05)	36.15 (23.57–50.13)	42.23 (29.58–54.89)	52.69 (40.78–64.59)	47.75 (37.54–57.97)
% Households with any LLINs or IRS (95% CI)	76.90 (70.39–83.40)	81.18 (75.97–86.38)	82.56 (78.02–87.11)	92.85 (89.83–95.87)	89.85 (85.26–94.44)	93.05 (89.89–69.23)	85.74 (79.86–91.63)	87.82 (83.99–91.64)	86.64 (80.43–92.85)
Case management (% children)									
% Children with fever in the past 2 weeks (95% CI)	20.82 (12.62–29.03)	25.18 (17.93–32.43)	24.28 (16.98–31.59)	16.51 (10.91–22.11)	12.12 (8.35–15.89)	17.23 (12.61–21.85)	12.87 (8.40–17.34)	12.83 (8.86–16.80)	18.35 (13.35–23.35)
Of children with fever, % taken for treatment at a public or private provider (95% CI)	60.09 (47.34–72.34)	67.34 (59.60–75.08)	69.20 (61.34–77.06)	60.16 (47.01–73.32)	67.09 (53.16–81.02)	63.11 (51.65–74.58)	60.19 (46.41–73.96)	68.22 (55.52–80.93)	64.43 (49.67–79.19)
Of children taken for treatment, % went to a CHW (95% CI)	9.16 (0.77–17.56)	9.58 (0.31–18.85)	16.46 (4.69–28.24)	10.82 (3.33–18.89)	16.98 (0.00–35.40)	19.48 (2.44–36.52)	12.31 (6.24–18.37)	10.96 (1.52–20.39)	21.88 (12.64–31.11)
% Households within 1.5 km of a malaria treatment provider (95% CI)	17.69 (0.00–37.79)	1.39 (0.21–2.57	18.54 (0.00–38.13)	38.29 (15.17–61.60)	39.57 (15.85–63.29)	49.15 (24.16–74.15)	42.43 (71.64–67.22)	39.81 (16.30–63.34)	48.15 (22.91–73.40)
Among children with fever and positive rapid diagnostic test, % treated with ACT reported (95% CI)	48.67 (55/113) (38.61–58.74)	57.69 (75/130) (46.48–68.90)	58.40 (73/125) (41.88–74.92)	55.00 (11/20) (34.46–75.54)	66.67 (8/12) (34.41–98.93)	33.33 (6/18) (0.00–74.28)	58.82 (10/17) (37.65–80.00)	38.46 (5/13) (0.00–81.74)	61.54 (8/13) (27.64–95.44)

CHW = community health worker; fMDA = focal MDA; IRS = indoor-residual spraying; LLIN = long-lasting insecticide-treated net; MDA = mass drug administration.

* Indoor-residual spraying coverage in 2014 is significantly different among MDA, fMDA, and control group (Rao–Scott χ^2^ = 6.0019, df = 2; *P* = 0.0497).

The proportion of children with reported fever in the past 2 weeks decreased from a mean of 23.4% across exposure groups at baseline to 15.4% after mass treatment rounds 1 and 2 and 14.6% after rounds 3 and 4 (Table 2, Rao–Scott χ^2^ = 24.72, df = 2; *P* < 0.0001). Seeking treatment at a public or private healthcare provider for children with fevers was high across all exposure groups and study years, ranging from 60.1% to 69.2%. Although the proportion of households within 1.5 km of a malaria treatment provider (CHW or health facility) did not differ across exposure groups, significantly more households were within this distance to a treatment provider in 2015 and 2016 than in the baseline of 2014 (Rao–Scott χ^2^ = 26.34, df = 2; *P* < 0.0001), which was due almost exclusively to increases in CHWs who started providing CCM for malaria in the latter half of 2014. For children with fevers, seeking care from a CHW increased from 2014 to the end of the trial in 2016, irrespective of the exposure group. Among children with a history of fever in the past 2 weeks who were found to be RDT positive at the time of the surveys, a significant proportion were reported to have been appropriately treated in the past 2 weeks with AL for that infection.

**Figure 3.**
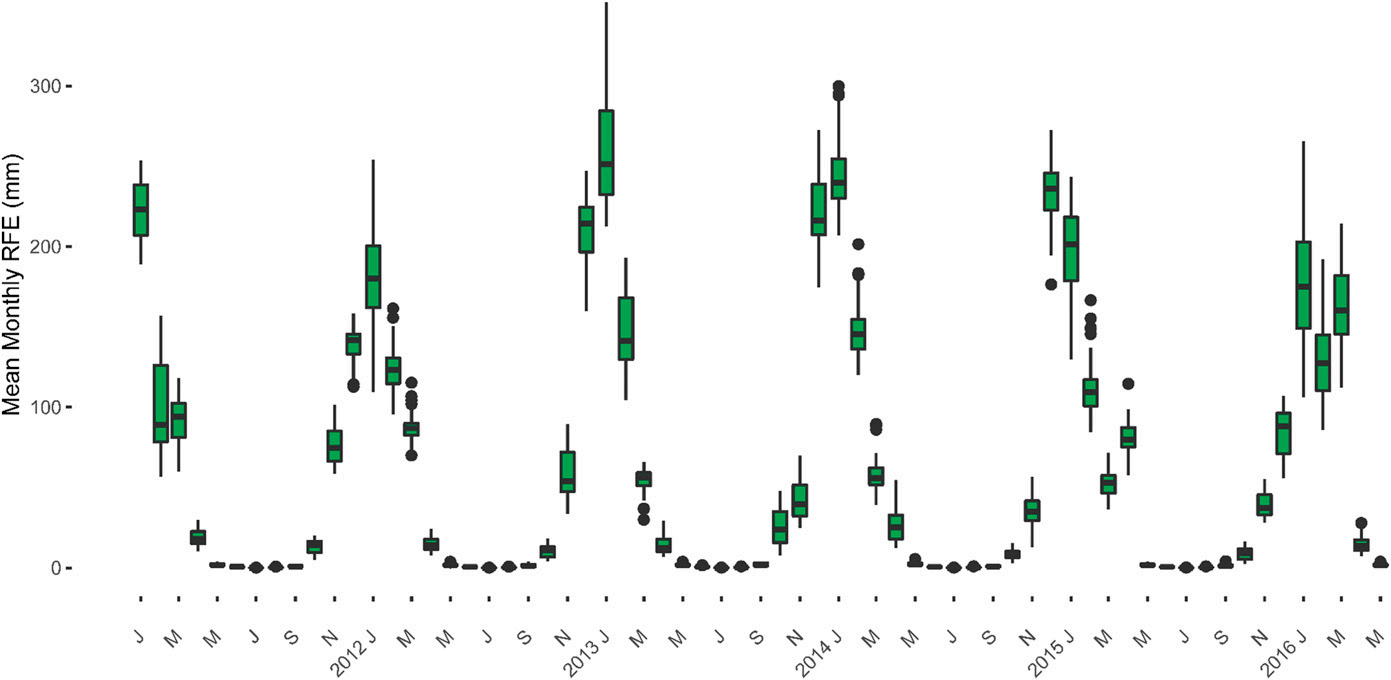
Total monthly rainfall, 2011–2016. This figure appears in color at www.ajtmh.org.

There was no difference between exposure groups in mean monthly total rainfall during the rainy seasons (January to April) during any of the study years (Figure 3). As reported previously,^3^ the mean monthly total rainfall during the rainy season in 2015 (during mass treatment rounds 1 and 2) was significantly lower than the rainfall during the 2014 rainy season at baseline. This trend continued in 2016 at the timing of mass treatment rounds 3 and 4, with mean monthly total rainfall during the rainy season falling to 105.8 mm (as compared with 1,030.3 mm in 2014 [*t*-test = 11.34, *P*-value < 0.001] and 116.4 mm in 2015 [*t*-test = 3.17, *P*-value = 0.0019]).

**Figure 4.**
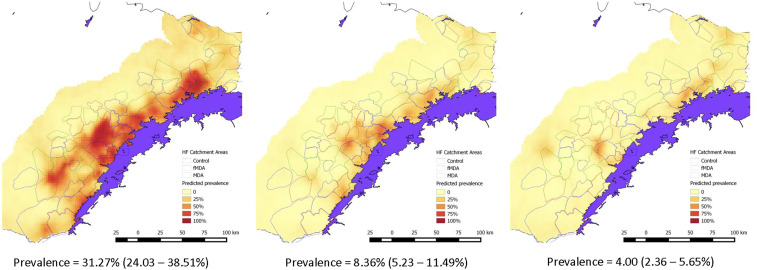
Spatial distribution of malaria parasite prevalence during peak transmission (April–May) in 2014–2016, from household surveys, Southern Province, Zambia.

Irrespective of the exposure group or transmission strata, the overall RDT parasite prevalence in children younger than 6 years during the peak malaria transmission season (April to May) declined precipitously from 31.3% in 2014 at baseline before the mass treatment rounds to 8.4% in 2015 after rounds 1 and 2 (a 73.2% decline), to 4.0% in 2016 after rounds 3 and 4 (an 87.2% decline, Figure 4). Parasite prevalence was highest in the lower elevation areas along Lake Kariba. As reported previously,^3^ only MDA in the low-transmission areas had a significant impact in reducing child parasite prevalence after the first two rounds (Table 3). Mass drug administration rounds 3 and 4 1 year later in the lower transmission areas led to a modest but not statistically significant further reduction in parasite prevalence compared with those that received the standard of care of the scaled intervention package (odds ratio = 0.58, 95% CI: 0.12–2.58). Overall, compared with populations receiving only the standard of care, those groups receiving four MDA rounds in areas of lower transmission experienced a significant decrease in the odds of a parasite infection in children by 72% after accounting for potential confounding factors (adjusted odds ratio = 0.28, 95% CI: 0.09–0.88). No significant impact of MDA in the higher transmission areas was observed, and no significant impact of fMDA was observed irrespective of transmission strata or the number of fMDA rounds.

**Table 3:** Impact of four rounds of mass treatment on malaria RDT-based parasite prevalence among children aged 3–70 months, as measured by household surveys during peak malaria transmission season (April–May) 2014–2016

		Baseline (April–May 2014)		Impact of mass treatment rounds 1–2 (April–May 2015)				Impact of mass treatment rounds 3–4 (April–May 2016)				Impact of all four mass treatment rounds (April–May 2015 and 2016 pooled)			
Exposure group		RDT+/*n*	Malaria infection prevalence, % (95% CI)†	RDT+/*n*	Malaria infection prevalence, % (95% CI)	Crude odds ratio vs. control at follow-up (95% CI)	Adjusted odds ratio at follow-up (95% CI)†	RDT+/*n*	Malaria infection prevalence, % (95% CI)	Crude odds ratio vs. control at follow-up (95% CI)	Adjusted odds ratio at follow-up (95% CI)†	RDT+/*n*	Malaria infection prevalence, % (95% CI)	Crude odds ratio vs. control at follow-up (95% CI)	Adjusted odds ratio at follow-up (95% CI)†
Lower transmission strata	MDA	42/545	7.71 (2.13–12.28)	2/372	0.54 (0.00–1.74)	0.19 (0.03–1.28)**	0.13 (0.02–0.92)*	3/392	0.77 (0.00–1.75)	0.58 (0.12–2.85)	0.53 (0.08–3.36)	5/764	0.65 (0.00–1.35	0.35 (0.11–1.12)**	0.28 (0.09–0.88)*
	fMDA	39/441	8.84 (1.88–15.80)	4/334	1.20 (0.00–2.79)	0.49 (0.11–2.29)	0.57 (0.13–2.50)	13/420	3.10 (0.60–5.59)	2.28 (0.66–7.90)	2.60 (0.57–11.83)	17/754	2.25 (0.61–3.89)	1.21 (0.48–3.02)	1.36 (0.57–3.30)
	Control	42/453	9.27 (3.11–15.44)	9/361	2.49 (0.21–4.78)	Reference	Reference	5/365	1.37 (0.00–3.00)	Reference	Reference	14/726	1.93 (0.47–3.39	Reference	Reference
Higher transmission strata	MDA	248/490	50.61 (35.40–63.38)	56/366	15.30 (4.68–25.92)	0.93 (0.26–3.35)	0.86 (0.25–3.04)	28/348	6.39 (2.18–10.60)	1.59 (0.44–5.77)	1.31 (0.49–3.49)	84/804	10.45 (4.42–16.47)	1.14 (0.38–3.43)	0.91 (0.40–2.12)
	fMDA	270/521	51.82 (36.02–67.63)	47/304	15.46 (5.08–25.84)	1.07 (0.30–3.86)	1.28 (0.36–4.60)	27/394	6.85 (0.00–14.36)	1.39 (0.38–5.11)	1.13 (0.40–3.19)	74/698	10.61 (3.41–17.79)	1.16 (0.38–3.52)	0.92 (0.40–2.12)
	Control	283/505	56.04 (38.60–73.48)	55/332	16.57 (7.87–25.26)	Reference	Reference	22/438	5.03 (0.00–10.41)	Reference	Reference	77/770	10.00 (3.80–16.20)	Reference	Reference

fMDA = focal MDA; MDA = mass drug administration; RDT = rapid diagnostic test.

† No statistically significant differences among MDA, fMDA, or control group prevalence at baseline.^3^

* *P* < 0.05.

** *P* < 0.10.

Among 2,230 individuals enrolled into the infection incidence cohort study just before the first mass treatment round, 92% completed at least 3 months of the first 6 months of follow-up and were included in the analysis. Across all arms, 92%, 83%, and 36% of individuals completed at least 6, 12, and 17 months of follow-up over the entire cohort period, respectively. A total of 593 PCR-detected infections were observed among the cohort followed for a total of 28,089 person-months of observation over the full 17-month follow-up period. Irrespective of the exposure group, the overall PCR infection incidence declined significantly over the 17-month follow-up period, from 29.0 infections per 1,000 during the first peak transmission season (January–May) in 2015 to 11.0 infections per 1,000 during the subsequent peak transmission season in 2016, a decline of 62% (Table 4). The overall cumulative PCR infection incidence over the follow-up period was lower in the MDA group than the standard of care in both the lower (3.6 versus 5.9 infections per 1,000, respectively) and higher (30.8 versus 47.9 infections per 1,000, respectively) transmission strata, although neither reduction was statistically significant. There was no significant difference between the fMDA and control group, irrespective of transmission strata or any period of the cohort follow-up.

**Table 4:** Cumulative *Plasmodium falciparum* infection incidence by PCR among individuals older than 3 months in cohort households followed monthly from January 2015–May 2016, by rainy and dry seasons

	Exposure group	Person-months	PCR positives	Cumulative incidence rate per 1,000 person-months (95 % CI)	Unadjusted incidence rate ratio vs. control (95% CI)	Adjusted incidence rate ratio vs. control (95% CI)
Lower transmission strata			Rainy season 1 after mass treatment rounds 1–2 (January–May 2015)			
	MDA	1,641	7	4.3 (1.7–8.8)	0.21 (0.03–1.34)	0.26 (0.04–1.90)
	fMDA	1,596	13	8.1 (4.3–13.9)	0.93 (0.19–4.53)	1.59 (0.34–7.55)
	Control	1,379	13	9.4 (5.0–16.1)	Reference	Reference
			Dry season after rounds 1–2 (June–November 2015)			
	MDA	1,682	7	4.2 (1.7–8.6)	0.49 (0.07–3.43)	0.75 (0.11–5.31)
	fMDA	1,790	4	2.2 (0.6–5.7)	0.58 (0.09–3.87)	1.01 (0.15–6.62)
	Control	1,545	6	3.9 (1.4–8.5)	Reference	Reference
			Rainy season 2 after mass treatment rounds 3–4 (December 2015–May 2016)			
	MDA	1,744	4	2.3 (0.6–5.9)	0.26 (0.03–2.00)	0.32 (0.04–2.62)
	fMDA	1,739	3	1.7 (0.4–5.0)	0.47 (0.07–3.35)	0.70 (0.10–5.02)
	Control	1,513	7	4.6 (1.9–9.5)	Reference	Reference
Higher transmission strata			Rainy season 1 after mass treatment rounds 1–2 (January–May 2015)			
	MDA	1, 499	51	34.0 (25.3–44.7)	0.44 (0.15–1.35)	0.38 (0.14–1.02)*
	fMDA	1,430	73	51.0 (40.0–64.2)	0.76 (0.26–2.25)	0.57 (0.21–1.56)
	Control	1,440	104	72.2 (59.0–87.5)	Reference	Reference
			Dry season after rounds 1–2 (June–November 2015)			
	MDA	1,596	75	47.0 (37.0–58.9)	1.09 (0.36–3.30)	1.40 (0.51–3.83)
	fMDA	1,518	64	42.2 (32.5–53.8)	1.26 (0.42–3.76)	1.52 (0.55–4.24)
	Control	1,560	72	46.2 (36.1–58.1)	Reference	Reference
			Rainy season 2 after mass treatment rounds 3–4 (December 2015–May 2016)			
	MDA	1,573	18	11.4 (6.8–18.1)	0.45 (0.14–1.50)	0.61 (0.19–1.93)
	fMDA	1,418	36	25.4 (17.8–35.1)	1.21 (0.38–3.78)	0.86 (0.27–2.77)
	Control	1,426	36	25.2 (17.7–35.0)	Reference	Reference

fMDA = focal MDA; MDA = mass drug administration. All standard errors of treatment effects are adjusted to account for the CRCT study design using a random effect at the cluster level in a negative binomial model. Adjusted model additionally included first month incidence, age, gender, household SES, vector control, rainfall, EVI, and elevation.

* *P* < 0.10.

Irrespective of the exposure group or transmission strata, confirmed malaria case incidence during the rainy season declined significantly from a baseline period from 2013 to 2014 (20.56 and 63.03 cases per 1,000 in the lower and higher strata, respectively) to 5.70 and 19.89 cases per 1,000 in the lower and higher strata, respectively, in 2015 and 2016 (Table 5). In the lower transmission strata, confirmed malaria case incidence declined by 37% more among those who received four rounds of MDA compared with those who received the scaled standard of care with no mass treatment (adjusted DiD IRR = 0.63, 95% CI: 0.49–0.80), after accounting for the baseline confirmed case incidence and potential confounding factors. This was consistent over the first two rounds (adjusted IRR = 0.59, 95% CI: 0.44–0.78) and rounds 3 and 4 (adjusted IRR = 0.71, 95% CI: 0.49–1.04). There was no observed benefit of MDA or fMDA in lowering confirmed malaria case incidence during the rainy season before and after the mass treatment periods in the higher transmission strata.

**Table 5:** Mean monthly confirmed malaria case incidence from the routine health information system

Exposure group	Monthly confirmed malaria case incidence rate per 1,000 catchment population				Adjusted difference-in-differences incidence rate ratio (95% CI)		
	Pre-intervention period (January–May 2013 and 2014)	Post-intervention period (January–May 2015) after rounds 1–2	Post-intervention period (January–May 2016) after rounds 3–4	Post-intervention period (January–May 2015 and 2016 pooled) after all four rounds	Pre-intervention vs. post-intervention 2015 after rounds 1–2	Pre-intervention vs. post-intervention 2016 after rounds 3–4	Pre-intervention vs. post-intervention 2015–2016 after all four rounds
Lower transmission strata							
MDA	17.79	3.89	2.99	3.43	0.59 (0.44–0.78)*	0.71 (0.49–1.04)	0.63 (0.49–0.80)*
fMDA	19.25	7.97	4.22	6.06	0.90 (0.70–1.15)	1.08 (0.77–1.52)	0.96 (0.77–1.19)
Control	24.33	8.32	5.27	6.80	Reference	Reference	Reference
Higher transmission strata							
MDA	59.20	15.24	7.24	11.18	0.92 (0.73–1.15)	1.03 (0.72–1.47)	0.94 (0.76–1.16)
fMDA	44.17	26.69	12.74	19.62	1.00 (0.80–1.25)	1.20 (0.84–1.70)	1.03 (0.84–1.27)
Control	78.90	47.35	8.25	27.50	Reference	Reference	Reference

fMDA = focal MDA; MDA = mass drug administration. Negative binomial model with random effect included at the health facility catchment area level, controlling for monthly total rainfall, EVI, and previous month’s case counts.

* *P* < 0.05.

## DISCUSSION

A community-randomized controlled trial was used to assess the impact of four rounds (during 2 years with two rounds per year) of MDA and fMDA with DHAP compared with a control group that received no mass treatment between 2014 and 2016 in 60 HFCAs in Southern Province, Zambia. The entire study area, including the control group, received a scaled standard of care intervention package consisting of high LLIN coverage (more than 77% household ownership), up to 50% household coverage with IRS Actellic 300CS, and improved surveillance and reporting through DHIS2. Importantly, the scaled intervention package included the use of CHWs to provide CCM that was scaled-up during the baseline period of 2014, resulting in vastly improved access to malaria diagnosis and treatment across the study communities. In this context, significant declines in all malaria health outcomes were observed over the course of the study, irrespective of the exposure group. Overall, parasite prevalence during the peak transmission season declined from 31.3% at baseline (2014) to 4.0% in 2016 (an 87% decline). Overall, cumulative PCR-based infection incidence measured in the cohort group declined by 62% during the peak transmission season in 2015 to 2016. In addition, irrespective of the exposure group, health management information system data showed that confirmed malaria case incidence declined during the peak transmission season from 42.2 per 1,000 before the trial to 12.9 per 1,000 in the 2015–2016 transmission seasons (a 69% decline).

As reported previously,^3^ children in communities that received the first two MDA rounds experienced a significant 87% reduction in the odds of a malaria parasite infection during the high transmission season in 2015 in areas of lower transmission, compared with children who received only the scaled standard of care without mass treatment. There was no observed additional benefit from MDA rounds 3 and 4 on child parasite prevalence during the peak transmission season in 2016, although parasite prevalence remained at < 1% in this group. However, across all four MDA rounds in areas of lower transmission, children who received MDA had a significant 78% (95% CI: 12–91% reduction) reduced odds of having a parasite infection during the high transmission seasons, as compared with the control group.

Similar to results for malaria parasite prevalence, the effect size for the four MDA rounds on PCR-confirmed infection incidence was greatest (IRR = 0.29) in the lower transmission strata. However, because of the overall decline in infection incidence over the course of the trial, there were very few PCR-confirmed infections observed in the lower transmission strata, and thus the power was quite low to detect statistical differences between different intervention and control groups in these areas. The only observed effect (marginally significant) of MDA on cumulative infection incidence was in the higher transmission strata during the peak transmission season following the first two rounds. Although there was no impact of MDA on the overall time to first infection over the full 17-month follow-up period, this may have been driven by the very low transmission during the dry season where few people in any exposure group became infected.^12^ However, over the first 6 months of the trial during the 2015 transmission season, individuals in communities that received the first two MDA rounds had a significant delay in the time to first infection (hazard ratio = 0.41, 95% CI: 0.18–0.91) in lower and higher transmission areas combined, after accounting for potential confounders.^12^

Data from confirmed malaria case incidence support the results from parasite prevalence and infection cohort incidence. Mass drug administration had a consistently larger impact in the lower transmission strata following the first two rounds, achieving a statistically significant 41% larger reduction in confirmed case incidence than the standard of care of receiving no mass treatment.

Our results in the lower transmission strata are similar to those observed in a recent cross-over MDA trial in four countries in the Greater Mekong Subregion.^14^ In this trial of three rounds of MDA-DHAP on top of a standard of care of improved access to case management and surveillance, *P. falciparum* prevalence was reduced from 5.1% at baseline to < 1% at follow-up, representing a significantly greater decline than the standard of care. However, the *P. falciparum* infection prevalence returned to near baseline levels 12 months later, likely as a result of reintroduction from surrounding areas.

Compared with the control group, fMDA did not have any observed impact on any study end points in this setting, irrespective of the number of rounds or the baseline transmission level. This was likely due to the overall lower population-level treatment coverage with DHAP that occurred by design in these areas, as compared with the MDA group, even though individuals testing positive by RDT who received DHAP may have benefited individually.

Vector control coverage during the study remained high (> 80% household coverage in 2015 and 2016). Moreover, the switch to Actellic 300CS for IRS in 2015 likely mitigated any pyrethroid or carbamate resistance in the study area,^15^ which has been reported in Zambia.^16^ However, although it is presumed that this level of vector control contributed to the overall decline in malaria transmission observed over the course of the study period, this trial was not designed to assess the impact of these interventions.

Improved access to prompt diagnosis and treatment of malaria, including the scale-up of CCM in the study area, appeared to have had a large impact on access to malaria diagnosis and treatment. Several observations support this conclusion. First, across all study years it was reported by mothers during the household surveys that among their children with a fever in the previous 2 weeks, more than 60% were taken for treatment to formal healthcare providers, including newly placed CHWs. Second, with the increase in numbers of trained CHWs providing CCM, with the majority of the increase occurring in the second half of the 2014 baseline year (there were only 187 trained CHWs in the trial area at the end of 2013, increasing to 423 by the end of 2014, resulting in a CHW to at-risk population of approximately 1/850), the proportion of households within 1.5 km of a malaria treatment provider significantly increased by approximately 3- to 4-fold from only 12.7% in the first half of 2014 before CHW scale-up, to 42.3% and 43.4% in 2015 and 2016, respectively. By 2016, as many as one in five children (21.9%) with fever taken for treatment were seen by a CHW in their area. Finally, the 2015 and 2016 household surveys showed that more than 50% of children were reported to have received treatment with AL for a recent case of malaria (those with a fever in the past 2 weeks and a positive RDT at the time of the survey). By contrast, a recent study found that among such children in large surveys across Africa, only 20% had received an artemisinin-based combination therapy for a recent bout of malaria defined the same way.^17^

In the context of lower rainfall during the 2015 and 2016 rainy seasons, we hypothesize that the increased coverage of effective vector control, which primarily reduces infectious bites (i.e., force of infection),^18–20^ along with much improved access to diagnosis and treatment, which shortens the duration of infection and infectivity,^21–26^ acted together to markedly decrease malaria transmission across the entire study area after the 2014 baseline year. The added value of MDA or fMDA could only be evaluated in this context of substantial improvement with the scaled intervention package. In control areas that received no mass treatment, child malaria parasite prevalence declined from 9.3% at the 2014 baseline to 1.9% in 2016 in areas of lower transmission, and from 56.0% to just 10.0% in areas of higher transmission. Comparative declines from baseline to 2016 among those who received MDA were 7.7–0.7% and 50.6–10.5% in areas of lower and higher transmission, respectively. In addition, providing more than 330,000 courses of DHAP over four rounds of MDA and fMDA in two-thirds of the study area may likely have lowered the overall parasite reservoir across the area that contributed indirectly to declines in control areas,^27^ possibly similar to the community effect seen in LLIN trials,^28^ as well as in a recently completed MDA trial in Myanmar.^29^

We observed the largest impact (effect size) of MDA during the first two rounds and in areas of lower transmission across study end points. This finding is consistent with a consensus of mathematical modeling studies.^30^

As noted previously for the results from the first two mass treatment rounds, results of this trial across all four rounds should be interpreted considering several potential limitations. First, we include here only a follow-up time of 2–3 months since the final round of mass treatment. We continue to monitor the longer term trends in malaria outcomes after the last mass treatment round; preliminary results suggest that there has been no rebound in malaria health outcomes as of May 2017, 15 months after the last MDA round, whereas the scaled standard of care intervention package has been maintained.^31^ Second, although the overall number of clusters in the trial was 60, comparison of each of the mass treatment approaches to the control group within each transmission strata only included 20 total clusters, rendering statistical power an issue as transmission declined more than expected in the control clusters. Third, the use of an intention-to-treat analysis could have resulted in misclassification of mass treatment exposure, which may have biased our results toward the null of no treatment effect. Fourth, we did not directly account for population mobility in our analyses, although overnight travel in the study area was estimated to be quite low.^32^ Fifth, the surveys relied on RDTs that may have overestimated malaria infection prevalence due to persistent antigenemia among those who were recently infected and treated before the surveys.^33^ However, there is no reason to believe this would have been different between exposure groups, and thus bias would have been limited. And finally, the use monthly cohort visits may have missed some infections that arose and were subsequently cleared between monthly visits.

In the context of a community-randomized controlled trial of four rounds of MDA or fMDA with DHAP in Southern Province, Zambia, all malaria health outcomes measured during the trial dropped precipitously from baseline, reaching pre-elimination levels across almost the entire study area by 2016, including in areas that had until recently experienced malaria parasite prevalence in excess of 50%.^5^ Our results show that MDA contributed significantly to the observed declines in some malaria outcomes during the peak malaria transmission seasons. We suggest that MDA should be considered for implementation in African settings only after the successful scale-up and maintenance of vector control, and only once very good access to malaria diagnosis and treatment has been established and maintained. It is in this context that MDA will likely have the most lasting impact on malaria transmission.

## Supplemental information tables

Supplemental materials

## References

[1] Zambia Ministry of Health, 2015 Zambia National Malaria Indicator Survey 2015. Lusaka, Zambia: Zambia Ministry of Health.

[2] Zambia Ministry of Health, 2017 NMEC, ed. National Malaria Elimination Strategic Plan 2017–2021: A Strategy to Move from Accelerated Burden Reduction to Malaria Elimination in Zambia (Brief). Lusaka, Zambia: Zambia Ministry of Health.

[3] Eisele TP 2016 Short-term impact of mass drug administration with dihydroartemisinin plus piperaquine on malaria in Southern Province Zambia: a cluster-randomized controlled trial. J Infect Dis 214: 1831–1839. 27923947 10.1093/infdis/jiw416 PMC5142084

[4] Eisele TP 2015 Assessing the effectiveness of household-level focal mass drug administration and community-wide mass drug administration for reducing malaria parasite infection prevalence and incidence in Southern Province, Zambia: study protocol for a community randomized controlled trial. Trials 16: 347. 26268804 10.1186/s13063-015-0862-3 PMC4535296

[5] Larsen DA Bennett A Silumbe K Hamainza B Yukich JO Keating J Littrell M Miller JM Steketee RW Eisele TP, 2015 Population-wide malaria testing and treatment with rapid diagnostic tests and artemether-lumefantrine in southern Zambia: a community randomized step-wedge control trial design. Am J Trop Med Hyg 92: 913–921. 25802434 10.4269/ajtmh.14-0347 PMC4426577

[6] Wei LJ Lachin JM, 1988 Properties of the urn randomization in clinical trials. Control Clin Trials 9: 345–364. 3203525 10.1016/0197-2456(88)90048-7

[7] Zambia Ministry of Health, 2014 Guidelines on Diagnosis and Treatment of Malaria in Zambia, 4th Edition Lusaka, Zambia: Zambia Ministry of Health.

[8] Silumbe K 2020 Assessment of the acceptability of testing and treatment during mass drug administration trial for malaria in Zambia using mixed methods. Am J Trop Med Hyg 103 (Suppl 2): 28–36. 10.4269/ajtmh.19-0663 PMC7416978 32618242

[9] Finn TP 2020 Treatment coverage estimation for mass drug administration for malaria with dihydroartemisinin-piperaquine in Southern Province, Zambia. Am J Trop Med Hyg 103 (Suppl 2): 19–27. 32618251 10.4269/ajtmh.19-0665 PMC7416979

[10] Finn TP 2020 Adherence to mass drug administration with dihydroartemisinin-piperaquine and *Plasmodium falciparum* clearance in Southern Province, Zambia. Am J Trop Med Hyg 103 (Suppl 2): 37–45. 32618267 10.4269/ajtmh.19-0667 PMC7416972

[11] Hayes RJ Bennett S, 1999 Simple sample size calculation for cluster-randomized trials. Int J Epidemiol 28: 319–326. 10342698 10.1093/ije/28.2.319

[12] Bennett A 2020 A longitudinal cohort to monitor malaria infection incidence during mass drug administration in Southern Province, Zambia. Am J Trop Med Hyg 103 (Suppl 2): 54–65. 32618245 10.4269/ajtmh.19-0657 PMC7416973

[13] Chalwe V Silumbe K Finn TP Hamainza B Porter T Kamuliwo M Kawesha EC Miller JM Steketee RW Eisele TP, 2017 Adverse event reporting from malaria mass drug administration rounds conducted in southern Zambia. Am J Trop Med Hyg 95: 487.

[14] von Seidlein L 2019 The impact of targeted malaria elimination with mass drug administrations on falciparum malaria in southeast Asia: a cluster randomised trial. PLoS Med 16: e1002745. 30768615 10.1371/journal.pmed.1002745 PMC6377128

[15] Chanda E Chanda J Kandyata A Phiri FN Muzia L Haque U Baboo KS, 2013 Efficacy of ACTELLIC 300 CS, pirimiphos methyl, for indoor residual spraying in areas of high vector resistance to pyrethroids and carbamates in Zambia. J Med Entomol 50: 1275–1281. 24843932 10.1603/me13041

[16] Choi KS 2014 Insecticide resistance and role in malaria transmission of *Anopheles funestus* populations from Zambia and Zimbabwe. Parasit Vectors 7: 464. 25293669 10.1186/s13071-014-0464-z PMC4197278

[17] Bennett A 2017 Population coverage of artemisinin-based combination treatment in children younger than 5 years with fever and *Plasmodium falciparum* infection in Africa, 2003–2015: a modelling study using data from national surveys. Lancet Glob Health 5: e418–e427. 28288746 10.1016/S2214-109X(17)30076-1 PMC5450656

[18] Lengeler C, 2004 Insecticide-treated bed nets and curtains for preventing malaria. Cochrane Database Syst Rev 2004: CD000363. 10.1002/14651858.CD000363.pub2 15106149

[19] Pluess B Tanser FC Lengeler C Sharp BL, 2010 Indoor residual spraying for preventing malaria. Cochrane Database Syst Rev 4: CD006657. 10.1002/14651858.CD006657.pub2 PMC6532743 20393950

[20] Gimnig JE 2003 Impact of permethrin-treated bed nets on entomologic indices in an area of intense year-round malaria transmission. Am J Trop Med Hyg 68: 16–22. 12749481

[21] Bousema JT 2006 Moderate effect of artemisinin-based combination therapy on transmission of *Plasmodium falciparum*. J Infect Dis 193: 1151–1159. 16544256 10.1086/503051

[22] Drakeley CJ Jawara M Targett GA Walraven G Obisike U Coleman R Pinder M Sutherland CJ, 2004 Addition of artesunate to chloroquine for treatment of *Plasmodium falciparum* malaria in Gambian children causes a significant but short-lived reduction in infectiousness for mosquitoes. Trop Med Int Health 9: 53–61. 14728607 10.1046/j.1365-3156.2003.01169.x

[23] Sutherland CJ Ord R Dunyo S Jawara M Drakeley CJ Alexander N Coleman R Pinder M Walraven G Targett GA, 2005 Reduction of malaria transmission to *Anopheles* mosquitoes with a six-dose regimen of co-artemether. PLoS Med 2: e92. 15839740 10.1371/journal.pmed.0020092 PMC1087200

[24] Griffin JT Ferguson NM Ghani AC, 2014 Estimates of the changing age-burden of *Plasmodium falciparum* malaria disease in sub-Saharan Africa. Nat Commun 5: 3136. 24518518 10.1038/ncomms4136 PMC3923296

[25] Okell LC 2014 Contrasting benefits of different artemisinin combination therapies as first-line malaria treatments using model-based cost-effectiveness analysis. Nat Commun 5: 5606. 25425081 10.1038/ncomms6606 PMC4263185

[26] Yukich J Briët O Bretscher MT Bennett A Lemma S Berhane Y, 2012 Estimating *Plasmodium falciparum* transmission rates in low-endemic settings using a combination of community prevalence and health facility data. PLoS One 7: e42861. 22936995 10.1371/journal.pone.0042861 PMC3425560

[27] Elliott RC Smith DL Echodu D, 2018 Medical and entomological malarial interventions, a comparison and synergy of two control measures using a Ross/MacDonald model variant and openmalaria simulation. Math Biosci 300: 187–200. 29655551 10.1016/j.mbs.2018.04.005 PMC6013649

[28] Hawley WA 2003 Community-wide effects of permethrin-treated bed nets on child mortality and malaria morbidity in western Kenya. Am J Trop Med Hyg 68: 121–127. 12749495

[29] Parker DM 2019 Potential herd protection against *Plasmodium falciparum* infections conferred by mass antimalarial drug administrations. Elife 8: e41023. 30990166 10.7554/eLife.41023 PMC6467567

[30] Brady OJ 2017 Role of mass drug administration in elimination of *Plasmodium falciparum* malaria: a consensus modelling study. Lancet Glob Health 5: e680–e687. 28566213 10.1016/S2214-109X(17)30220-6 PMC5469936

[31] Eisele TP 2020 Impact of four rounds of mass drug administration with dihydroartemisinin-piperaquine implemented in Southern Province, Zambia. Am J Trop Med Hyg 103 (Suppl 2): 7–18. 10.4269/ajtmh.19-0659 PMC7416977 32618247

[32] Porter T 2017 Assessing associations between recent travel and malaria parasite prevalence during a mass drug administration campaign in southern Zambia. Am J Trop Med Hyg 95: 282.

[33] Keating J Miller J Bennett A Moonga H Eisele T, 2009 *Plasmodium falciparum* parasite infection prevalence from a household survey in Zambia using microscopy and a rapid diagnostic test: implications for monitoring and evaluation. Acta Trop 112: 277–282. 19682968 10.1016/j.actatropica.2009.08.011

