# Supplementary material for: Impact of Four Rounds of Mass Drug Administration with Dihydroartemisinin–Piperaquine Implemented in Southern Province, Zambia
Source: Am J Trop Med Hyg. 2020 Jul 2;103(2 Suppl):7–18. doi: 10.4269/ajtmh.19-0659 (PMC7416977; doi:10.4269/ajtmh.19-0659)
Supplement: Supplementary file 1 [file tpmd190659.SD1.pdf]

Supplemental information

SI Table 1 Age-based dosing guidelines used for administering DHAp (Signma Tau)

Age	Weight (kg)	DHAP (40/320mg)		
	(kg)	pill count per day	mg 40	mg 320
0–3 months	<5			
3–12 months	8	0.5	2.50	20.00
1 year	10	1	4.00	32.00
2	11	1	3.64	29.09
3	15	1	2.67	21.33
4	15	1	2.67	21.33
5	18	1	2.22	17.78
6	21	1	1.90	15.24
7	23	1	1.74	13.91
8	25	2	3.20	25.60
9	28	2	2.86	22.86
10	30	2	2.67	21.33
11	35	2	2.29	18.29
12	39	2	2.05	16.41
13	45	2	1.78	14.22
14	50	3	2.40	19.20
≥15 years	>50	3	<2.4	<19.2
		**AVG mg/kg/day**	**2.57**	**20.57**

SI Table 2. Baseline characteristics of intervention and control households obtained from the parasite survey in April-May 2014

**Characteristic**	**MDA** (n = 1,047 children / 857 households) **(95 % CI)**	**fMDA** (n = 985 children / 850 households) **(95 % CI)**	**Control** (n = 976 children / 866 households) **(95 % CI)**
% Children by age included for parasite testing (in years)			
3 month – 1 year	12.70 (9.81 – 15.60)	11.98 (9.98 – 14.03)	12.81 (10.62 – 14.50)
1	17.96 (15.89 – 20.02)	17.87 (14.73 – 21.01)	15.68 (13.56 – 17.79)
2	15.85 (13.99 – 17.72)	17.36 (15.18 – 19.54)	17.52 (13.45 – 21.59)
3	15.76 (13.44 – 18.07)	17.46 (14.95 – 19.97)	17.62 (15.29 – 19.96)
4	17.67 (14.50 – 20.84)	17.87 (15.28 – 20.45)	19.06 (17.34 – 20.77)
5	20.06 (17.23 – 22.88)	17.46 (14.70 – 20.23)	17.32 (14.08 – 20.55)
Sex of children included for parasite testing (% male)	48.14 (44.90 – 51.37)	50.86 (46.44 – 55.28)	52.25 (49.00 – 55.51)
% Children included for parasite testing by household wealth quintile			
1 - Poorest	32.66 (23.63 – 41.70)	21.73 (15.63 – 27.82)	27.97 (20.05 – 35.89)
2	19.10 (13.38 – 24.82)	25.69 (20.78 – 30.59)	19.06 (14.41 – 23.70)
3	20.73 (15.77 – 25.68)	19.29 (14.21 – 24.37)	21.21 (16.16 – 26.26)
4	17.86 (12.64 – 23.08)	16.65 (12.20 – 21.10)	18.95 (14.38 – 23.53)
5 - Least poor	9.65 (6.04 – 13.25)	16.65 (9.41 – 23.89)	12.81 (6.99 – 18.63)

*Significantly different with $p < 0.05$
